# Coding of Childhood Psychiatric and Neurodevelopmental Disorders in Electronic Health Records of a Large Integrated Health Care System: Validation Study

**DOI:** 10.2196/56812

**Published:** 2024-05-14

**Authors:** Jiaxiao M Shi, Vicki Y Chiu, Chantal C Avila, Sierra Lewis, Daniella Park, Morgan R Peltier, Darios Getahun

**Affiliations:** 1 Department of Research and Evaluation, Kaiser Permanente Southern California, Pasadena, CA, United States; 2 Department of Psychiatry, Jersey Shore University Medical Center, Neptune, NJ, United States

**Keywords:** autism, autism spectrum disorder, ASD, attention deficit hyperactivity disorder, ADHD, disruptive behavioral disorders, DBD, anxiety disorders, AD, major depression disorder, MDD, autistic, coding, neurodevelopmental, psychiatric, electronic health record, electronic health records, validation, accuracy, mental health, emotional, behavior, behaviors, behavioral, disorder, disorders, pediatric, pediatrics, paediatric, infant, paediatrics, infants, infancy, baby, babies, neonate, neotnates, neonatal, toddler, toddlers, child, children, hospital, hospitals

## Abstract

**Background:**

Mental, emotional, and behavioral disorders are chronic pediatric conditions, and their prevalence has been on the rise over recent decades. Affected children have long-term health sequelae and a decline in health-related quality of life. Due to the lack of a validated database for pharmacoepidemiological research on selected mental, emotional, and behavioral disorders, there is uncertainty in their reported prevalence in the literature.

**Objectives:**

We aimed to evaluate the accuracy of coding related to pediatric mental, emotional, and behavioral disorders in a large integrated health care system’s electronic health records (EHRs) and compare the coding quality before and after the implementation of the *International Classification of Diseases, Tenth Revision, Clinical Modification* (*ICD-10-CM*) coding as well as before and after the COVID-19 pandemic.

**Methods:**

Medical records of 1200 member children aged 2-17 years with at least 1 clinical visit before the COVID-19 pandemic (January 1, 2012, to December 31, 2014, the *ICD-9-CM* coding period; and January 1, 2017, to December 31, 2019, the *ICD-10-CM* coding period) and after the COVID-19 pandemic (January 1, 2021, to December 31, 2022) were selected with stratified random sampling from EHRs for chart review. Two trained research associates reviewed the EHRs for all potential cases of autism spectrum disorder (ASD), attention-deficit hyperactivity disorder (ADHD), major depression disorder (MDD), anxiety disorder (AD), and disruptive behavior disorders (DBD) in children during the study period. Children were considered cases only if there was a mention of any one of the conditions (yes for diagnosis) in the electronic chart during the corresponding time period. The validity of diagnosis codes was evaluated by directly comparing them with the gold standard of chart abstraction using sensitivity, specificity, positive predictive value, negative predictive value, the summary statistics of the *F*-score, and Youden *J* statistic. κ statistic for interrater reliability among the 2 abstractors was calculated.

**Results:**

The overall agreement between the identification of mental, behavioral, and emotional conditions using diagnosis codes compared to medical record abstraction was strong and similar across the *ICD-9-CM* and *ICD-10-CM* coding periods as well as during the prepandemic and pandemic time periods. The performance of AD coding, while strong, was relatively lower compared to the other conditions. The weighted sensitivity, specificity, positive predictive value, and negative predictive value for each of the 5 conditions were as follows: 100%, 100%, 99.2%, and 100%, respectively, for ASD; 100%, 99.9%, 99.2%, and 100%, respectively, for ADHD; 100%, 100%, 100%, and 100%, respectively for DBD; 87.7%, 100%, 100%, and 99.2%, respectively, for AD; and 100%, 100%, 99.2%, and 100%, respectively, for MDD. The *F*-score and Youden *J* statistic ranged between 87.7% and 100%. The overall agreement between abstractors was almost perfect (κ=95%).

**Conclusions:**

Diagnostic codes are quite reliable for identifying selected childhood mental, behavioral, and emotional conditions. The findings remained similar during the pandemic and after the implementation of the ICD-10-CM coding in the EHR system.

## Introduction

Children and adolescents are particularly vulnerable to chronic mental and behavioral conditions because their brain continues to develop. Childhood mental and behavioral disorders, including autism spectrum disorder (ASD), attention-deficit hyperactivity disorder (ADHD), disruptive behavior disorders (DBD), anxiety disorder (AD), and major depressive disorder (MDD), are common neurological disorders and are on the rise in recent decades [1-4]. Affected children and adolescents are subjected to long-term negative health and social consequences [5,6], leading to significant health care costs and public health burden [7,8]. Therefore, accurately estimating their incidence and prevalence is important to guide policy-making, resource allocation, and implementation of different intervention programs.

Trends in mental and behavioral disorders are difficult to examine using routinely collected data and often are difficult to compare across studies because of differences in case ascertainment methods. Therefore, reported incidence and prevalence rates vary widely across studies [9], and the accuracy of case ascertainment has been challenged by researchers [10,11]. This problem is further complicated by the accuracy of coding after the mandatory introduction of the *International Classification of Diseases, Tenth Revision, Clinical Modification (ICD-10-CM)* coding systems to classify diagnoses and procedures in the United States, which occurred on October 1, 2015 [12]. The *ICD-10-CM* provides increased specificity and detail for many health conditions [12], including childhood psychiatric and neurodevelopmental conditions. Many health care providers adopted the electronic health record (EHR) enacted by the Health Information Technology for Economic and Clinical Health Act (HITECH Act) in 2009 [13,14].

The Kaiser Permanente Southern California (KPSC)–integrated EHR data provide researchers with important health information to perform pharmacoepidemiological studies of children’s behavioral, medical, and psychiatric conditions, including examining the public health impact of childhood psychiatric and neurodevelopmental conditions; however, there is uncertainty over the accuracy of the clinical diagnosis codes requiring validation before their use. Therefore, the objective of this study was to perform an EHR review on the validity of diagnosis codes during 3 time points (*ICD-9-CM* and *ICD-10-CM* coding and before and after the COVID-19 pandemic) for ascertaining psychiatric and neurodevelopmental conditions in a large socioeconomically diverse pediatric population aged 2-17 years [15]. The validation for the pre- and post–COVID-19 pandemic is important to assess how much the diagnosis coding has been impacted by increasing the use of virtual visits (telephone and video-assisted encounters).

## Methods

### Study Setting

This study was conducted using data on member children extracted from the KPSC EHR. The KPSC health care system provides services to over 4.8 million members in 15 hospitals and 234 medical offices throughout southern California. Mental health services are provided to member patients by qualified providers at in- and outpatient psychiatric care facilities in KPSC settings. Although most members receive their care at KPSC hospitals and <10% use contracting hospitals, all diagnostic, procedural, and pharmacy records are captured and maintained by the KPSC EHR since its full implementation in 2008. The sociodemographic characteristics of KPSC members closely reflect the California population [15].

### Ethical Considerations

This study was conducted with approval by the KPSC Institutional Review Board (IRB# 13114). Informed consent was waived, as the study was low risk and strictly involved the use of internal EHR data, accessible only to authorized personnel when needed.

### Study Design and Sample Selection

Data for this validation study were obtained retrospectively from children who were members of the KPSC health care system during three distinct time periods: (1) January 1, 2012, through December 31, 2014; (2) January 1, 2017, through December 31, 2019; and (3) January 1, 2021, through December 31, 2022. We carefully selected the 3 time periods to investigate the medical coding accuracy of selected psychiatric and neurodevelopmental conditions encompassing both the *ICD-9-CM* and *ICD-10-CM* periods as well as the pre- and post–COVID-19 pandemic eras. To be included in the validation study cohort for each time period, children must have been enrolled in the KPSC health care system for at least 1 year during the corresponding time period at specific age ranges varying by condition (5-17 years of age for ADHD, 2-17 years of age for ASD, and 3-17 years of age for the other 3 conditions—AD, MDD, and DBD). Children may present with signs and symptoms of the specified conditions very early in life; however, for this study, we used reported and reliable lower age groupings for ASD and ADHD diagnoses [16,17] and widely published age goupings for anxiety and depressive disorders [18,19]. Furthermore, at least 1 clinical visit, including virtual visits, in each corresponding time period was required.

In the KPSC system, diagnosis codes for clinical visits (eg, hospitalization, outpatient office visits, and emergency department visits) from all KPSC facilities are extracted from EHRs and entered into a structured database by professional medical coders from the clinical data management team. In this validation study, coding-based outcomes of interest were ascertained using these clinical diagnosis codes within the 3 specified time periods (Multimedia Appendix 1 presents the *ICD-9-CM* and *ICD-10-CM* codes).

For each of the investigated conditions, we randomly sampled 40 cases according to the following strata: (1) those without a diagnosis (No-Dx) and (2) those with a diagnosis (Dx). Thus, records for a total of 1200 individual children were selected. The accuracy for each of the 2 strata, with or without documented diagnostic records, was expected to be around 85%. A sample size of 120 per diagnostic condition would provide less than a 5% one-sided margin for a 90% CI of the accuracy for each stratum.

### Diagnosing ASD, ADHD, DBD, and MDD

The KPSC system has an integrated framework for in- and outpatient as well as emergency department encounter services. During the child’s visit to any of these facilities, the practitioner has access to the child’s diagnoses, but often the diagnosis of ASD, ADHD, and DBD is made in an outpatient setting. The following criteria were used to diagnose and code ASD, ADHD, and DBD within the KPSC setting: (1) a Child Behavior Checklist must be filled out by parents and teachers to describe the child’s behavioral and emotional problems and (2) a clinical interview must be performed by a qualified mental health professional. In a preliminary study conducted for this project, 96% of children with ASD, ADHD, and DBD were found to have had their conditions diagnosed by KPSC child and adolescent psychiatrists, developmental and behavioral pediatricians, child psychologists, and neurologists consistent with the diagnostic criteria from the *Diagnostic and Statistical Manual of Mental Disorders-Fifth Edition* (*DSM-V*). Diagnosis of the remaining 4% was confirmed upon membership, as these cases had been previously diagnosed outside the KPSC system [20,21].

The diagnosis of depression disorder, in the KPSC system, was based on the US Preventive Services Task Force on screening for depression in children and adolescents recommendation [22]. The KPSC system uses the Patient Health Questionnaire (PHQ-9) and the PHQ-9 modified for Adolescents (PHQ-A). If a patient’s score on the PHQ-9 or PHQ-A does not seem to accurately reflect observed clinical symptoms, *DSM-V* criteria are recommended for diagnosis.

To ascertain the neurodevelopmental conditions investigated in this study, we relied on systemwide clinical diagnoses made by experts in the field as mentioned above.

### Chart Abstraction Process

Trained research associates (abstractors) reviewed EHRs for documentation of a diagnosis (yes/no) of each condition under investigation for the selected sample of children during the study period. Children were considered to have the disorder in the presence of documented evidence of that condition noted in the chart during the corresponding time period of investigation. To ensure data quality and consistency of chart reviews between the 2 abstractors, a total of 180 cases, stratified by the 2 strata (Dx and No-Dx), were randomly selected for reabstraction (90 per abstractor and 36 per condition). There was a total of 4 possible responses for each chart reviewed case, as follows: diagnosis “Yes,” diagnosis “No,” “Unable to ascertain due to blocked notes,” and “Unable to ascertain due to insufficient notes.” The abstractors based their responses on the information they were able to ascertain from the clinical notes. For example, due to the sensitive nature of psychiatric diagnoses, some progress notes may have been blocked; therefore, if the abstractors could not ascertain the diagnosis due to blocked notes, it was coded as such. Similarly, if the abstractors could not ascertain a diagnosis due to a lack of documentation or notes in the KPSC system with underused care (eg, outside claims data), the record was coded as “Unable to ascertain due to insufficient notes.”

The results of this assessment informed our full chart review process of the 1200 medical records as mentioned above. In other words, we excluded those claims data records from the random selection of the validation sample. Furthermore, during chart review, records in which the abstractors encountered blocked notes and were therefore unable to make a determination were flagged. These flagged records (n=38, 3%) were replaced with another randomly selected record for chart review from the same strata. Potential cases that were still unclear in the clinical use records were adjudicated by the study investigator with expertise in the field (DG). The child psychiatric and neurodevelopmental condition cases abstracted through this process served as the gold standard.

### Child Characteristics

Characteristics for KPSC member children included age (2-5, 6-11, and 12-17 years), sex (male and female), race/ethnicity (categorized as non-Hispanic White, non-Hispanic Black, Hispanic, Asian/Pacific Islander, other/multiple, and unknown), median household income in US dollars (<30,000; 30,000-49,999; 50,000-69,999; 70,000-89,999; and ≥90,000), and insurance type (Medicaid, commercial through employment, private/individual, and other).

We obtained the characteristics of all children of the state of California residents during the same time periods using publicly available data posted on the Centers for Disease Control and Prevention Wonder website [3]. Both the KPSC EHR and the Centers for Disease Control and Prevention (Wonder) sources provided information on child characteristics, including age and race/ethnicity. Data on median household income were estimated based on census tracts for KPSC patients.

### Statistical Analysis

We described the characteristics of our study population between those with and without the conditions of interest. Furthermore, we investigated how representative the KPSC pediatric population is compared to the state of California pediatric population during the entire study period using frequency distributions. For the purpose of comparison with the California children population, the age of the children for the KPSC population was evaluated based on the date of randomly selected clinical visits for each child during the entire study period.

The agreement between the 2 abstractors was evaluated with the interrater reliability assessment (κ statistic) by using the initial 180 chart reviews. We compared findings from the manual chart review of the 1200 cases, set as the gold standard, with corresponding diagnosis records for he *ICD-9-CM* and *ICD-10-CM* coding periods as well as before and after the COVID-19 pandemic through sensitivity, specificity, positive predictive value (PPV), and negative predictive value (NPV). These performance measurements were reported as weighted percentages with corresponding 95% CIs using normalized sampling weights (*W*), as defined below:


$$W_{ij}=\frac{\#\;in\;stratum\;j}{120\times \left(\#total\;study\;population\;i\right)},\;i=1\;to\;5,j=1,2$$

where i is for each of the 5 conditions and j is for the corresponding two strata (Dx and No-Dx) within each condition. To evaluate the overall performance, we also reported the summary statistics of *F*-score and Youden *J* statistic, which are composite measurements of sensitivity and PPV (*F*-score) or sensitivity and specificity (*J* statistic).

All analyses were conducted using SAS statistical software (version 9.4; SAS Institute, Inc).

## Results

An overview of the patient characteristics for our sample, study population, and the state of California pediatric population are shown in Table 1. Overall, 2,283,931 children from all KPSC hospitals and medical offices were obtained during the 3 time periods. Compared with the state of California pediatric population, KPSC had similar distributions on the children’s sex but had slightly higher percentages of children for ages 2-5 years (25.6% for KPSC vs 24.7% for the state of California) and 6-11 (37.8% for KPSC vs 37.4% for the state of California). In addition, KPSC had slightly lower percentages of children identified as non-Hispanic White (24.5% vs 28.5%) or Asian/Pacific Islander (10.0% vs 13.1%). However, this could be partially attributed to the larger percentage of unknown, missing, or multiple race/ethnicity in the KPSC group (6.0% vs 0.5%, respectively). Overall, the KPSC pediatric population in this study was a good representation of the entire state of California’s pediatric population. Our sample of 1200 children across the 5 conditions was broadly representative of the KPSC study population with respect to age, sex, and race/ethnicity. However, children in the older age groups were slightly oversampled. The overall κ between our 2 abstractors was 95%.

**Table 1.** Characteristics of the study cohort and the state of California pediatric population aged 2-17 years (2012-2014, 2017-2019, and 2021-2022).

Characteristics		Chart reviewed		Pediatric population	
		Yes (n=1200)	No (n=2,282,731)	KPSC^a^ (n=2,283,931)	California state^b^ (n=48,566,041)
**Age**^c^ **(years), n (%)**					
	2-5	198 (16.5)	584,657 (25.6)	584,855 (25.6)	11,975,915 (24.7)
	6-11	473 (39.4)	863,628 (37.8)	864,101 (37.8)	18,169,267 (37.4)
	12-17	529 (44.1)	834,446 (36.6)	834,975 (36.6)	18,420,859 (37.9)
**Child’s sex, n (%)**					
	Female	536 (44.7)	1,117,357 (48.9)	1,117,893 (48.9)	23,755,606 (48.9)
	Male	664 (55.3)	1,165,374 (51.1)	1,166,038 (51.1)	24,810,435 (51.1)
**Race/ethnicity, n (%)**					
	Non-Hispanic White	322 (26.8)	558,254 (24.5)	558,576 (24.5)	13,818,677 (28.5)
	Non-Hispanic Black	90 (7.5)	180,348 (7.9)	180,438 (7.9)	3,056,231 (6.3)
	Hispanic	622 (51.8)	1,178,606 (51.6)	1,179,228 (51.6)	25,096,264 (51.7)
	Asian/Pacific Islander	98 (8.2)	228,687 (10.0)	228,785 (10.0)	6,344,897 (13.1)
	Unknown/other/multiple	68 (5.7)	136,836 (6.0)	136,904 (6.0)	249,972 (0.5)
**Household income (US $), n (%)**					
	<30,000	24 (2.0)	58,366 (2.6)	58,390 (2.6)	—^d^
	30,000-49,999	256 (21.3)	481,524 (21.1)	481,780 (21.1)	—
	50,000-69,999	336 (28.0)	609,418 (26.7)	609,754 (26.7)	—
	70,000-89,999	260 (21.7)	482,178 (21.1)	482,438 (21.1)	—
	≥90,000	323 (26.9)	649,109 (28.4)	649,432 (28.4)	—
	Missing	1 (0.1)	2136 (0.1)	2137 (0.1)	—
**Insurance type, n (%)**					
	Medicaid	302 (25.2)	534,606 (23.4)	534,908 (23.4)	—^d^
	Commercial	843 (70.3)	1,642,058 (71.9)	164,2901 (71.9)	—
	Private	49 (4.1)	93,984 (4.1)	94,033 (4.1)	—
	Other	6 (0.5)	12,083 (0.5)	12,089 (0.5)	—

a KPSC: Kaiser Permanente Southern California; the sample is based on the KPSC data from the electronic health records (2012-2014, 2017-2019, and 202-2022).

b Data are from the natality information of Centers for Disease Control and Prevention website [3].

c Starting age is different for each condition.

d Median household income and insurance type information are not available for the California state data.

Table 2 shows the distribution and their sample sizes for the two strata (Dx and No-Dx) among our entire study population and by the 3 time periods. Based on these comparisons against the chart review results, the performance measurements of our EHRs are shown in Table 3. The weighted sensitivity, specificity, PPV, and NPV for each of the 5 conditions were as follows:

- ADHD: sensitivity 100%, specificity 99.9%, PPV 99.2%, and NPV 100%
- ASD: sensitivity 100%, specificity 100%, PPV 99.2%, and NPV 100%
- MDD: sensitivity 100%, specificity 100%, PPV 99.2%, and NPV 100%
- AD: sensitivity 87.7%, specificity 100%, PPV 100%, and NPV 99.2%
- DBD: sensitivity 100%, specificity 100%, PPV 100%, and NPV 100%

The corresponding *F*-score and Youden *J* statistic were 99.6% and 99.9% for ADHD, 99.6% and 100% for ASD, 99.6% and 100% for MMD, 93.4% and 87.7% for AD, and 100% and 100% for DBD, respectively. Results were similar across the *ICD-9-CM* and *ICD-10-CM* coding time periods as well as the before and after the COVID-19 pandemic.

**Table 2.** Frequencies of study population and chart review results by diagnosis codes. Condition-specific row percentages may not sum to 100% due to rounding.

Condition and diagnosis status			Study population				Sample		
			Overall, n	Before COVID-19 pandemic, n (%)		After COVID-19 pandemic (2021-2022), n (%)	Charts abstracted, n		Normalized sampling weight
				2012-2014	2017-2019		Total	Confirmed cases	
**DBD** ^a^									
	Dx^b^		41,351	11,787 (28.5)	14,907 (36.1)	14,657 (35.5)	120	120	0.0001601556
	No-Dx^c^		2,110,254	697956 (33.1)	755,726 (35.8)	656,572 (31.1)	120	0	0.0081731777
**Anxiety**									
	Dx		120,484	26039 (21.6)	49,720 (41.3)	44,725 (37.1)	120	120	0.0004666439
	No-Dx		2,031,121	683704 (33.7)	720,913 (35.5)	626,504 (30.9)	120	1	0.0078666894
**ASD** ^d^									
	Dx		47,441	9953 (21.0)	16,355 (34.5)	21,133 (44.6)	120	119	0.000173097
	No-Dx		2,236,490	741,058 (33.1)	802,267 (35.9)	693,165 (31.0)	120	0	0.0081602363
**MDD** ^e^									
	Dx		43,919	7846 (17.9)	19,036 (43.3)	17,037 (38.8)	120	119	0.0001701017
	No-Dx		2,107,686	701,897 (33.3)	751,597 (35.7)	654,192 (31.0)	120	0	0.0081632316
**ADHD** ^f^									
	Dx		108,717	35,668 (32.8)	40,791 (37.5)	32,258 (29.7)	120	119	0.0004826384
	No-Dx		1,768,413	587,686 (33.2)	630,533 (35.7)	550,194 (31.1)	120	0	0.0078506949

a DBD: disruptive behavior disorders.

b Dx: with confirmed diagnosis codes in electronic data.

c No-Dx: without confirmed diagnosis codes in electronic data.

d ASD: autism spectrum disorder.

e MDD: major depressive disorder.

f ADHD: attention deficit hyperactivity disorder.

**Table 3.** Weighted performance measurements for childhood psychiatric and neurodevelopmental disorders by data sources before and after the implementation of the *International Classification of Diseases, Ninth/Tenth Revisions, Clinical Modification* codes in the Kaiser Permanente Southern California (KPSC) system in 2015 (ample size=1200) and before and after the COVID-19 pandemic.

Disease and periods		Weighted performance measurements					
		Sensitivity (95% CI)	Specificity (95% CI)	PPV^a^ (95% CI)	NPV^b^ (95% CI)	*F*-score	Youden *J* statistic
**DBD** ^c^							
	Overall	100 (100-100)	100 (100-100)	100 (100-100)	100 (100-100)	100	100
2012-2014	100 (100-100)	100 (100-100)	100 (100-100)	100 (100-100)	100	100
2017-2019	100 (100-100)	100 (100-100)	100 (100-100)	100 (100-100)	100	100
2021-2022	100 (100-100)	100 (100-100)	100 (100-100)	100 (100-100)	100	100
**Anxiety**							
	Overall	87.7 (63.4-100)	100 (100-100)	100 (100-100)	99.2 (97.5-100)	93.4	87.7
2012-2014	100 (100-100)	100 (100-100)	100 (100-100)	100 (100-100)	100	100
2017-2019	100 (100-100)	100 (100-100)	100 (100-100)	100 (100-100)	100	100
2021-2022	70.4 (11.3-100)	100 (100-100)	100 (100-100)	97.5 (92.5-100)	82.6	70.4
**MDD** ^d^							
	Overall	100 (100-100)	100 (99.9-100)	99.2 (97.5-100)	100 (100-100)	99.6	100
2012-2014	100 (100-100)	100 (100-100)	100 (100-100)	100 (100-100)	100	100
2017-2019	100 (100-100)	100 (100-100)	100 (100-100)	100 (100-100)	100	100
2021-2022	100 (100-100)	99.9 (99.8-100)	97.5 (92.5-100)	100 (100-100)	98.7	99.9
**ASD** ^e^							
	Overall	100 (100-100)	100 (99.9-100)	99.2 (97.5-100)	100 (100-100)	99.6	100
2012-2014	100 (100-100)	99.9 (99.8-100)	97.5 (92.5-100)	100 (100-100)	98.7	99.9
2017-2019	100 (100-100)	100 (100-100)	100 (100-100)	100 (100-100)	100	100
2021-2022	100 (100-100)	100 (100-100)	100 (100-100)	100 (100-100)	100	100
**ADHD** ^f^							
	Overall	100 (100-100)	99.9 (99.8-100)	99.2 (97.5-100)	100 (100-100)	99.6	99.9
2012-2014	100 (100-100)	100 (100-100)	100 (100-100)	100 (100-100)	100	100
2017-2019	100 (100-100)	100 (100-100)	100 (100-100)	100 (100-100)	100	100
2021-2022	100 (100-100)	99.8 (99.5-100)	97.5 (92.5-100)	100 (100-100)	98.7	99.8

a PPV: positive predictive value.

b NPV: negative predictive value.

c DBD: disruptive behavior disorders.

d MDD: major depressive disorder.

e ASD: autism spectrum disorder.

f ADHD: attention deficit hyperactivity disorder.

## Discussion

### Principal Findings

This validation study was performed to determine the accuracy of clinical diagnosis codes in ascertaining childhood psychiatric and neurodevelopmental cases using data abstracted from the EHR of a large integrated health care system serving a demographically diverse patient population. To our knowledge, the accuracy of data on studied conditions using clinical diagnostic codes has not been validated using EHR data. Furthermore, the extent to which the transition of *ICD-9-CM* to the *ICD-10-CM* coding system as well as the pre- and post–COVID-19 pandemic eras have impacted the ascertainment of the studied behavioral and developmental conditions is unclear. Our study showed that within a large integrated health system, there is strong agreement between the diagnosis codes (*ICD-9-CM* or *ICD-10-CM*) and the patients’ conditions, including ASD, ADHD, DBD, AD, and MDD, both before and after the COVID-19 pandemic eras.

In recent years, EHRs have become important data sources for various epidemiological study designs investigating potential associations between exposures and outcomes that have become standard among researchers and health care providers as part of the American Recovery and Reinvestment Act of 2009 (specifically the HITECH Act) [14]. Although the EHR has become an important information management and care delivery system tool ensuring quality of care by providing access to comprehensive treatment-related data, its validity and completeness for conducting pharmacoepidemiological studies have been challenged by many due to how information is coded [23]. In the KPSC health care system, the process of using data coding and coding rules of the medical diagnoses and procedures recorded in patients’ health records is performed by trained medical coders from the clinical data management team. Individual diagnostic conditions in the EHR need to be evaluated critically for accuracy and consistency. Furthermore, whether the accuracy of case ascertainment has been impacted by the introduction of the *ICD-10-CM* coding system as well as the effect of the COVID-19 pandemic on the quality of data capture needs to be investigated. Therefore, we performed this validation study to evaluate (1) the accuracy of pediatric mental, emotional, and behavioral disorders identified in EHRs and (2) the coding quality before and after the implementation of the *ICD-10-CM* codings as well as before and after the COVID-19 pandemic.

The findings of this study suggest that the validity of EHR data for the identification of childhood mental, behavioral, and emotional conditions is quite strong and the transition from the *ICD-9-CM* coding system to the *ICD-10-CM* coding system as well as coding during the COVID-19 pandemic had minimal impact on the overall accuracy of case ascertainment.

The main strength of this study is the large chart abstraction conducted to assess the validity and reliability of childhood mental, behavioral, and emotional disorders case ascertainment using EHR data extracted from a large integrated health care system. The EHR system database provides an opportunity for neurodevelopmental outcome investigation with a high degree of validity of case ascertainment for epidemiological studies, in addition to current developments by others using natural language processing algorithms [24-27]. This validation study, comprised of a demographically diverse southern California population, is likely generalizable to health care settings with similar EHR database systems. Although the overall agreement between our abstractors was almost perfect (κ=95%), a potential limitation of this study was the use of medical record abstractors who were not blinded to the source of the data. However, a previous study that evaluated the agreement between masked and unmasked medical record abstraction reported no impact of bias in case ascertainment [28]. A further potential limitation is the fact that we had some records with blocked notes that needed to be replaced because of incompleteness in ascertaining the conditions of interest. However, in close examination, we found that the blocked notes were only 38 (3%) out of the 1200 cases, which we believe will have minimal impact on the overall analysis. In addition, we did not consider oversampling of the older age strata in the summarized performance metrics. Considering that the older age group could be given a more accurate diagnosis coding, the actual concordance of the electronic coding might be slightly lower than what we observed in this study.

### Conclusions

Our findings suggest that childhood mental, behavioral, and emotional disorders are reliably coded in the EHRs and can be used for pharmacoepidemiological studies. Furthermore, the completeness of data remained similar during the pre- and postpandemic eras and after the implementation of the *ICD-10-CM* coding in the EHR system.

## Supplementary material

10.2196/56812 Multimedia Appendix 1 *International Classification of Diseases, Ninth/Tenth Revisions, Clinical Modification* diagnostic codes to ascertain mental, emotional, and behavioral disorders.

## Abbreviations

AD: anxiety disorder
ADHD: attention deficit hyperactivity disorder
ASD: autism spectrum disorder
DBD: disruptive behavioral disorders
DSM-V: Diagnostic and Statistical Manual of Mental Disorders-Fifth Edition
Dx: with confirmed diagnosis codes in electronic data
EHR: electronic health record
HITECH: Health Information Technology for Economic and Clinical Health
*ICD-10-CM*: *International Classification of Diseases, Tenth Revision, Clinical Modification*
*ICD-9-CM*: *International Classification of Diseases, Ninth Revision, Clinical Modification*
KPSC: Kaiser Permanente Southern California
MDD: major depression disorder
No-Dx: without confirmed diagnosis codes in electronic data
NPV: negative predictive value
PHQ: Patient Health Questionnaire
PHQ-A: Patient Health Questionnaire modified for Adolescents
PPV: positive predictive value
