# Supplementary material for: Coding of Childhood Psychiatric and Neurodevelopmental Disorders in Electronic Health Records of a Large Integrated Health Care System: Validation Study
Source: JMIR Ment Health. 2024 May 14;11:e56812. doi: 10.2196/56812 (PMC11107768; doi:10.2196/56812)
Supplement: Multimedia Appendix 1 [file mental-v11-e56812-s001.docx]

**Appendix Table.**ICD9/10 diagnostic codes to ascertain mental, emotional and behavioral disorders.

| **Condition** | **ICD-9-CM Code** | **ICD-10-CM Code** |
| --- | --- | --- |
| **ADHD** | 314.00, 314.01, 314.1, 314.2, 314.8, 314.9 | F90.0, F90.1, F90.2, F90.8, F90.9 |
| **ASD** | 299.0, 299.00, 299.01,  299.10, 299.11, 299.80, 299.81, 299.90, 299.91 | F84.0, F84.5, F84.8, F84.9 |
| **MDD** | 296.20, 296.21, 296.22, 296.23, 296.24, 296.25, 296.26, 296.30, 296.31, 296.32, 296.33, 296.34, 296.35, 296.36 | F32.0, F32.1, F32.2, F32.3, F32.4,  F32.5, F32.9, F33.0, F33.1, F33.2,  F33.3, F33.40, F33.41, F33.42, F33.9 |
| **AD** | 300.00, 300.01, 300.02,  300.09, 300.20, 300.21,  300.22, 300.23, 300.29,  300.3, 301.4, 309.21,  313.0, 313.23, 313.89 | F40.x, F41.0, F41.1, F41.3, F41.8,  F41.9, F42, F42.2, F42.3, F42.4,  F42.8, F42.9, F93.0, F94.0, F94.1 |
| **DBD** | 312.00, 312.10, 312.20,  312.81, 312.82, 312.89,  312.9, 313.81 | F34. 81, F91.0, F91.1, F91.2, F91.3, F91.8, F91.9, F92.0 |

Abbreviation: DBD, disruptive behavior disorders; MDD, major depressive disorder; ASD, autism spectrum disorder; ADHD, attention deficit hyperactivity disorder
